# Management of the poultry red mite, *Dermanyssus gallinae*, using silica-based acaricides

**DOI:** 10.1007/s10493-020-00541-y

**Published:** 2020-09-08

**Authors:** Christian Ulrichs, Young Jong Han, Magdi T. Abdelhamid, Inga Mewis

**Affiliations:** 1 Faculty of Life Sciences, Division Urban Plant Ecophysiology, Humboldt-Universität zu Berlin, Lentzeallee 55, 14195 Berlin, Germany; 2 Botany Department, National Research Centre, 33 EL Bohouth St. Dokki, Cairo, 12622 Egypt

**Keywords:** Chicken, Poultry, Ectoparasite, Pest control, Physical acaricides, Synthetic amorphous silicon dioxide

## Abstract

Four silica-based acaricides were examined in laboratory tests for their effectiveness against poultry red mite, *Dermanyssus gallinae*. All acaricides resulted in 100% mite mortality. Two groups of active ingredients could be differentiated. The products Silicosec^®^ and Ewazid^®^, based on naturally occurring diatomaceous earth (DE), killed 100% of adult *D. gallinae* within 48 h exposure time. The time to kill 50% of the mites (LT_50_) was calculated to be 31.7 and 34.9 h, respectively. The other two products, containing aggregates and agglomerates of pyrogenic synthetic amorphous silicon dioxide as active ingredients, killed the mites in a significantly shorter time: LT_50_ was 6.3 h for the liquid product Fossil Shield^®^ Instant White and 11.8 h for the powdery product Fossil Shield 90.0 White. This is more remarkable as the quantities of active ingredients used for the DE treatments were several folds higher. The effectiveness of all tested products was also shown in practical tests. A professional company treated five chicken houses on one farm in the Berlin–Brandenburg region with the test products, three houses with Fossil Shield Instant White and one each with Ewazid and Silicosec. Over a period of 46 weeks after stocking, the mite development in the houses was assessed. Only in one of the houses, treated with Fossil Shield Instant White, the mite population remained permanently low. In two houses treated with Fossil Shield Instant White, small mite colonies appeared in week 36, which were controlled by a follow-up spot treatment in week 41. In the houses treated with DE, the first mite colonies appeared 12 weeks after stocking. The number increased continuously over the experimental period and in week 31 after stocking there were clearly visible colonies (2–3 cm diameter) and the first mites could also be detected on the chicken eggs. At this time both houses were treated again with a follow-up spot-treatment, which only led to a slight improvement in one house and to a stabilization of the infestation in the other house. In week 41, large mite colonies were detected in both houses. A spot treatment at this point was ineffective in reducing the infestation. The tests showed faster acaricidal action of the products with the synthetic active ingredients compared to the natural DE-based products. This matches the shorter killing times under laboratory conditions. The experiments in a commercial chicken farm showed that it is possible to control the mite population for a period of 46 weeks by using physically effective SiO_2_-based products. These products are therefore an effective alternative to the use of chemical acaricides.

## Introduction

The poultry red mite, *Dermanyssus gallinae*, is the most important pest in egg-laying hens in many parts of the world (Sparagano et al. 2009; Wang et al. 2010). Even if in some countries other mite species are more important (Wales et al. 2010), in many countries (including Germany) *D. gallinae* tends to predominate. In the European Union (EU) most of the commercial egg-laying facilities are infested with *D. gallinae*. For example, Flochlay et al. (2017) reported, that 83% of the EU farms are infested by *D. gallinae*. Fiddes et al. (2005) reported an infestation rate of 62% of egg-laying hen farms for the UK and Mul (2016) reported infestation rates up to 94% for The Netherlands, Germany and Belgium.

Several authors report a relationship between *D. gallinae* infestation and hen mortality, under severe infestations causing hens to become anemic (Kilpinen et al. 2005; Wojcik et al. 2000). The economic costs associated with losses in hen productivity and hen mortality has been estimated to be 130 million Euro per year in the EU alone (Sparagano et al. 2009). Other negative effects of infestations include reduced animal welfare, reduced egg quality, and lower bird weight (Chauve 1998).

Research concerning all aspects of *D. gallinae* has increased in recent years. Commonly the pest is controlled using synthetic acaricides with over 35 compounds tested (Sparagano et al. 2014). However, only few products are licensed in the EU. At the same time, residues in laying hens prove that there are more products applied (Marangi et al. 2012). In many countries, resistance to synthetic acaricides in several chemical groups, mainly carbamates and pyrethroids, has been reported (Beugnet et al. 1997; Marangi et al. 2009; Nordenfors et al. 2001). In a British survey published in 2004, more than 60% of all farms surveyed had experienced acaricide resistant infestations (Guy et al. 2004). The use of synthetic products is further limited due to stricter legislation leading to a reduced number of products in the EU in recent years (Sparagano et al. 2014). Meanwhile, few new synthetic acaricides like the phoxim-based product ByeMite (Bayer, Germany) are being developed against *D. gallinae*. Whereas initial reports stated a high efficacy (Keita et al. 2006; Meyer-Kühling et al. 2007), resistance has already been assumed in Poland (Zdybel et al. 2011). Consumer awareness and the demand for pesticide-free products are pushing the search for alternative management strategies away from synthetics.

Silica-based products (silicas) contain mainly silicon dioxide (SiO_2_) as a biocidal substance. They are among the remaining options to control *D. gallinae* (Kilpinen and Steenberg 2009; Maurer et al. 2009). Both natural and synthetic silica products are applied for pest control. Synthetic products contain exclusively amorphous SiO_2_ whereas natural products have been mainly based on diatomaceous earth (DE) which contains a small crystalline fraction. Amorphous silica is ‘generally considered safe’ by the US Federal Drug Administration, and amorphous silica nanoparticles are often used as ‘negative controls’ in toxicity studies of nanocrystalline quartz (Ghiazza et al. 2010). The efficacy of silica products against arthropods varies widely (Mucha-Pelzer et al. 2008). Studies show that correlations exist between the insecticidal/acaricidal efficacy and the absorption capacity of the particles (Faulde et al. 2006; Schulz et al. 2014), chemical composition (Ulrichs et al. 2008), particle size (Arnaud et al. 2005), and specific surface (Islam et al. 2010). In the early days of application when mainly natural SiO_2_ products (DE) were used, they were surface-treated to make them water-repellent and therefore more effective. Today we see increasing use of purely synthetic compounds coming onto the market.

The purpose of the present study was (1) to compare new synthetic silica-based products with products based on DE, (2) to identify the most effective materials in laboratory tests, and (3) to indicate their efficacy under field conditions in a commercial hen house.

## Materials and methods

### Test substances

All test products are commercially available from various producers. Table 1 shows products tested in laboratory experiments. Silicosec^®^ (100 g powder in 600 ml water), Ewazid^®^ Silgur F46 (100 g powder in 600 ml water), and Fossil Shield^®^ Instant White (100 g powder in 800 ml water) were prepared as recommended by the producer. Fossil Shield 90.0 White was applied as a powdery dry material.

**Table 1:** Test products, mean active substances and calculated mean amount for applications

Product [CAS number]	Dried product (mg/dish)^d^	Active substance (mg/dish)^d^	Product (g/m^2^)	Active substance (g/m^2^)
Fossil Shield^®^ 90.0 White^a^ [68909-20-6]	112	5.028	18.63	0.836
Fossil Shield^®^ Instant White^b^ [68909-20-6]	247	17.78	41.07	2.96
Silicosec^®^ fluid^c^ [61790-53-2]	440	440	73.17	73.17
Ewazid^®^ Silgur F46^c^ [61790-53-2]	258	258	42.91	42.91

^a^4.5% and ^b^7.2% active ingredient pyrogenic, synthetic amorphous SiO_2_ (aggregates and agglomerates in nanoform); ^c^100% diatomaceous earth as active ingredient

^d^Surface of the dish was 60.13 cm^2^

### Laboratory experiments

The experiments were carried out under controlled climatic conditions (25 °C, 80% relative humidity) in a Conviron MTR26 plant growth cabinet (Winnipeg, Canada) at Humboldt-Universität zu Berlin, Germany. All experiments were conducted with mites in open plastic Petri dishes (9 cm diameter). By using open dishes, the climate corresponded to the conditions set in the plant growth cabinet.

The inside base of the Petri dishes used were ground to roughen the surface for better adhesion of the test substances. Grinding residues were removed with the help of compressed air. To prepare the fluid products, the powdery test substances were mixed in water. Mixing was carried out using a Collomix DLX 152 m and a standard drill. The test substances were stirred into water and the sample taken for application into the empty Petri dishes. The powdery test substance was transferred into the Petri dishes with the aid of a brush and weighed in. An attempt was made to achieve an even coating of the surface by gently shaking the dish.

Six Petri dishes were coated for each of the test substances and the four dishes with the most uniform coating were selected. Each Petri dish was counted as a replication. The liquid products were applied with the aid of a Birchmeier Spray-Matic 1.25 N, a full cone nozzle and a pressure of 3 bar from a distance of approximately 30–40 cm. The liquid-coated dishes were stored at room temperature for drying. After drying, excess material was removed from the edge of the dishes and the trays were weighed to calculate the quantities used. The edge of the Petri dishes was coated with non-stick substance (provided by Bein, Eiterfeld, Germany). This coating prevents mites from leaving the open Petri dish. The dry dishes were adapted to the climatic conditions in the experiment 48 h before mites were introduced. Untreated Petri dishes served as control.

All experiments were executed in dark conditions, which is the natural condition of a nocturnal food search for *D. gallinae.* Mites were not fed during the laboratory experiments. A mixed population of *D. gallinae* was collected in a chicken house 1 day before the experiment. In the chicken house mite colonies were found especially near the approach plates, at corners and edges near the resting places of the hens. Until treatment the mites were adapted to the experimental climate conditions in the same plant growth cabinet where the experiments took place. Mites were then transferred to the Petri dishes in the laboratory directly before the test. For this purpose, a stick was held in the vessel with mites. The mites migrated up the stick, which meant that only vigorous mites were tested. The migrating mites were transferred to the prepared test Petri dishes by tapping the rod vigorously. Therefore, all developmental stages which could hold on to the stick took part in the experiment. These are predominantly protonymphal and deutonymphal stages and adult mites. Small larvae, freshly hatched, would not be able to hold as well to the stick compared to older stages, and eggs were also thus excluded. Altogether, a minimum of 30 individual developed mites were transferred into each Petri dish.

Mortality rates were controlled under a stereoscopic microscope over a period of 48 h. To facilitate control, the mites were moved to one side of the dish by holding the Petri dish at an angle and tapping on the side. This facilitated counting and enabled a comparison of mite conditions, as vigorous mites can adhere well to most surfaces. However, as soon as they are only ‘partially vital’, this characteristic is lost. During the control, the mites were only declared ‘dead’ if there was no more self-motion visible. Some of them remained motionless for a long time and then began to walk again. A good indication of the death of the animals is the curvature of the legs towards the corpus, which is typical for arthropods, and a sunken and noticeably less shiny body. Furthermore, it was observed that the digestion process of the mites always slowed down during exposure and finally came to a standstill. In unclear physical condition, the Petri dish was slightly tapped, which prompted living poultry red mites to move.

### Field experiments

To test the efficacy under real conditions, a practical trial in 2017 compared the synthetic silica-based Fossil Shield Instant White with two DE products Silicosec and Ewazid Silgur-F46. The application was carried in five wet-cleaned and disinfected houses of a commercial laying hen farm in Brandenburg, Germany. Four houses were 1000 m^2^, one 950 m^2^. The density of chickens for housing was 15 hens/m^2^ accessible area. Chickens of the Lohmann brown breed were kept at 16–32 °C and 65–80% RH. Light was given according to the course of a day and in accordance with the management guide by Garrelfs et al. (2016). The animals were kept in floor management in so-called multi-tier aviaries. The tiers provide living space at several levels, allowing hens to disperse across levels. This way the tiers increase the total accessible area (max. 4 tiers according to EU Directive), enabling higher stocking densities compared to conventional floor housing. The aviaries were made of metal. Wood shavings were used as bedding material and renewed twice a week.

Treatment of the houses took place on 9 November 2016 and 3 days later the chickens were housed. The occurrence of mites in the houses was assessed at 12, 27, 31, 36, 41, and 46 weeks after treatment. No mite traps were used to monitor the population development, but fixed sample sites were examined. We decided against the use of traps because although they are very suitable for indicating the presence of mites, they are less suitable for quantification. Observations in the past have often shown that the mites stayed near but not in the traps, therefore not reflecting the intensity of the infestation.

Five sample sites were selected for each house segment. One house segment is approx. 3 m long. In the houses there are 50 segments each in two frames, every 4th segment was evaluated. With the help of an illuminated telescope mirror, it was possible to check areas difficult to access for mite infestation. In particular, the undersides of the approach plates, cross and longitudinal beams of the house, perches, connecting pieces to drinking lines as well as the entire head on both sides of the house were examined. In addition, the manure belt, ventilation systems, and the pipes of the perch rods, some of which were not closed at the head, were inspected by video endoscopy. The mite development was evaluated on a scale from 0 to 5 (Table 2) over a period of 46 weeks (Fig. 2).

**Table 2:** Score to estimate mite population in houses

0	No mites
1	Individual mites, without colonies (usually beginning at the head parts of the house)
2	Approx. 5–7 colonies the size of a pinhead (on headboards and 10% of the house segments [front plates, very hidden])
3	> 10 colonies the size of a thumbnail in 100% of the segments, visible on anterior and middle plates and supports
4	> 15 well visible colonies (2–3 cm diameter) in the complete house, beginning behavioral abnormality in hens, sporadic mites when collecting eggs for the first time in the morning
5	Large mite colonies on the complete house, mite odor, anemia, behavioral abnormalities, mites on the eggs, increased hen mortality

In the field tests, the liquid products were applied according to the manufacturer’s specifications by means of a diaphragm pump system and spray nozzles for every layer of the structure. For the product Silicosec 1200 l was applied in the stable on 950 m^2^. The product Ewazid Silgur F46 was applied with 1050 l on a surface of 1000 m^2^ and Fossil Shield Instant White with 880 l per 1000 m^2^ surface. The application system was designed in such a way that all the levels of the house were coated with test material. After drying, before stocking the hens, a dense dry product film remained on the surfaces. During stocking with hens this coating wears out. When the first mite colonies (score 2, Table 2) reappeared, a specific treatment of the mite population took place in these houses. This selective spot-treatment took place with a simple backpack sprayer. Only spots with visible mite colonies were treated.

In two houses treated with DE (1 and 2), a first follow-up treatment was carried out after 31 weeks. In houses 1, 2, 3 and 5 the follow-up treatment was carried out after 41 weeks. Only in house 4 no significant colonies were identified and therefore no follow-up treatment was carried out.

### Statistical evaluation

The statistics software package SPSS v.25 was used. A General Linear Model was used to search for deviations between the mean values after 24 h exposure time. Tukey’s honestly significant difference (HSD) test was used to separate mean values among the test substances for the examination after 24 h (α = 0.05). The time at which 50% and 90% of the mites died after treatment (LT_50_ and LT_90_) was determined by probit analysis. Abbott (1925) correction of the data was waived in the lab experiments, but the mortality rate in the controls were reported. The field trials could not be evaluated statistically due to a lack of repetition for Silicosec and Ewazid.

## Results

### Laboratory experiments

**Fig. 1:**
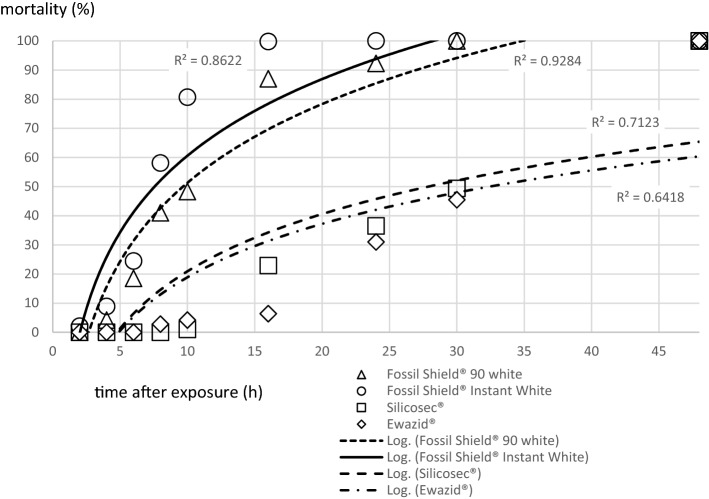
*Dermanyssus gallinae* cumulative mortality over time after treatment with each of four test substances

Twenty-four h after treatment with both Fossil Shield formulations mite mortality reached 100% under laboratory conditions (Fig. 1). Treatment with Silicosec and Ewazid caused an average mortality after 24 h of 36.5 and 31%, respectively; however, 100% of all mites were killed within 48 h after treatment (Fig. 1).

All treatments increased mortality within 24 h significantly (Fig. 1). There was no difference in efficacy within active ingredient group (DE vs. pyrogenic, synthetic amorphous, surface-treated SiO_2_) (Table 3). However, the synthetic silica products (Fossil Shield) were more effective after 24 h of exposure compared to the natural products (Ewazid and Silicosec) (Table 3). Control mortality was 3.85% after 24 h of exposure and 4.8% after 48 h.

**Table 3:** Cumulative mortality (%) for *Dermanyssus gallinae* after 24 and 48 h of exposure to each of four silica-based acaricides under laboratory conditions

Exposure time (h)	Control	Fossil Shield 90 white	Fossil Shield Instant White	Silicosec	Ewazid
24	3.85a	92.3c	100c	36.5b	31.0b
48	4.80a	100b	100b	100b	100b

Different letters following means within a row indicate significant differences among products (Tukey’s HSD: p < 0.05)

The comparison of the LT_50_ and LT_90_ values showed differences in the efficacy of the test substances (Table 4). The most effective test substance was Fossil Shield Instant White with LT_50_ = 6.4 h and LT_90_ = 9.5 h. Fossil Shield 90.0 White killed 50% after 11.8 h and 90% after 18.5 h. For Silicosec LT_50_ was 31.7 h and LT_90_ 52.4 h, whereas for Ewazid LT_50_ was 34.9 h and LT_90_ 55.9 h.

**Table 4:** Lethal time (h) after which 50% (LT_50_) or 90% (LT_90_) of all mites have been killed (95% confidence intervals in parentheses), determined by probit analysis, for the test substances

Product	LT_50_	LT_90_
Fossil Shield 90.0 White	11.821 (10.881–13.879)	19.247 (18.548–21.082)
Fossil Shield Instant White	6.357 (5.077–7.634)	9.396 (8.027–12.414)
Silicosec	31.706 (25.720-44.086)	52.416 (41.098–76.449)
Ewazid	34.988 (28.447–46.288)	55.874 (42.264–79.101)

### Experiments in houses with egg-laying hens

**Fig. 2:**
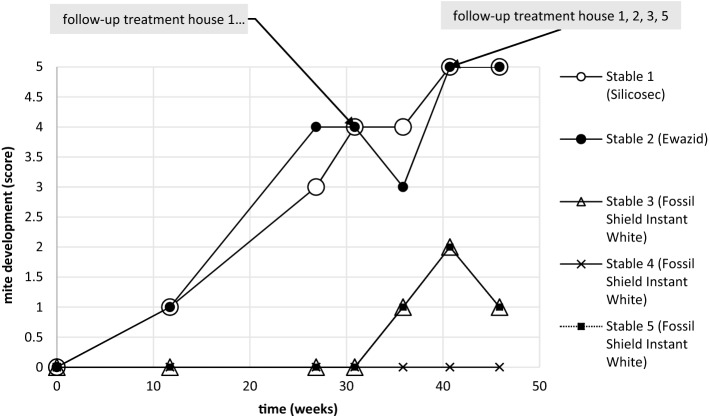
Mite development (based on mite presence scores; see Table 2) over 46 weeks in five houses (stables), each treated with one silica product, after chickens were housed (9 November 2016)

The development of mite populations in the houses showed large differences depending on the treatment. In the two houses treated with DE-based products, the population increased over the trial period of 46 weeks (Fig. 2). In the three houses where Fossil Shield Instant White was used, there was no population increase during first 31 weeks; however, after 31 weeks, population growth was observed in two houses (Fig. 1).

As the experiments were conducted in commercial production systems, farmers did not want to lose productivity and have therefore treated houses where mite population increased within the observation period. A spot follow-up treatment was applied in houses 1 and 2 with Silicosec and Ewazid, respectively. In house 1 the population did not further increase until week 41, in house 2 the mite population first decreased of the, but recovered by week 41.

In two houses (3 and 5) initially treated with Fossil Shield Instant White at week 41 the population decreased to a lower score until the last observation date (week 46).

## Discussion

With increasing resistance of *D. gallinae* to synthetic acaricides and changes in legislation and production practices, it is likely that *D. gallinae* will pose an ever-increasing threat to global poultry production. The fastest and easiest way to control mites in the henhouse is to use chemical agents. However, resistances against acaricides (Murano et al. 2009) and the chemical residue after treatment (Marangi et al. 2012) is viewed critically. Resistances have been reported in many countries and for different groups of active ingredients (Beugnet et al. 1997; Nordenfors et al. 2001). Marangi et al. (2012) tested 45 hens from three farms and found carbaryl residues in over 40% of their organ and tissue samples, and permethrin residue was detected in nearly 2% of their samples. Carbaryl is banned by the EU and permethrin not licensed.

For this reason, it is important to search for products that are highly effective while being user-friendly and more environmentally friendly. Physical treatments using chemically inert dusts could make a significant contribution here. Among the best studied inert substances are DE (Mewis and Ulrichs 2001; Islam et al. 2010; Badii et al. 2013), but also kaolin (Mullens et al. 2012) and synthetic silicates (Maurer et al. 2009; Luis et al. 2019) have been used against the poultry red mite *D. gallinae*. Numerous substances are sold in powder form, but for some years now liquid applications have also been increasingly finding their way into practice. The mechanism of action is mainly due to the absorption of lipids from the epicuticle of the mites, leading to desiccation (Mewis and Ulrichs 2001).

### Comparison of products

Under laboratory conditions, both Fossil Shield products with the active substance pyrogenic, synthetic amorphous, surface-treated SiO_2_, lead to a significantly faster killing of *D. gallinae* than the products with DE as active ingredient, Silicosec and Ewazid (Tables 3, 4). However, the tests do not allow conclusions to be drawn about dose-dependent mortality, as various amounts of active ingredients have been applied. Still, the difference between the two active ingredients and their efficacy becomes more apparent, if the quantities of active substance used are considered. For Silicosec and Ewazid the amount of active substance applied was much higher that for the Fossil Shield products (Table 1). In contrast, Fossil Shield products consist of an active substance and a filler. If the filler is removed, only 0.8 mg m^−2^ active substance was applied for 90.0 White and 2.9 mg m^−2^ active substance for Instant White. In contrast the DE product did not contain any filler.

Numerous studies have shown that the mortality rate for DE is not very high within a short period after application at high relative humidity (Schulz et al. 2014). Due to microclimatic differences, sub-optimal application of the product to surfaces, and the ability of the mites to absorb food and thus counteract water loss (Schulz et al. 2014), the necessary exposure times in field experiments to kill all mites is longer than under optimal laboratory conditions.

It must be noted that due to the way the mites were introduced into the Petri dishes, no eggs were introduced. Products based on DE have only a minor effect on eggs (Schulz et al. 2014). It is therefore more important that the products exhibit a long-term effect.

### Field experiments and practical implications

All products effectively reduced *D. gallinae* populations in experiments conducted in commercial hen houses. However, there were differences in the effectiveness under these conditions. Here, it is not so much the knock-down effect and rapid effectiveness as in the laboratory tests that play a role, but the long-term control of the mites. Only one of the houses treated with Fossil Shield Instant White had a permanently low mite population over 46 weeks. In two of the houses, small mite colonies first appeared in week 36, which were controlled by follow-up spot treatment in week 41. In contrast to this, the houses treated with Silicosec and Ewazid (both with DE as active ingredient), the first mite colonies appeared 12 weeks after stocking. The number increased continuously over the experimental period and in week 31 after stocking there were clearly visible colonies (2–3 cm in diameter) and the first mites could also be detected on the chicken eggs. At this time both houses were treated again with a follow-up spot-treatment, which only led to a slight improvement in one house and to a stabilization of the infestation in the other house. In week 41 large mite colonies were detected in both houses. A spot treatment was not effective in reducing the infestation.

Despite the higher amount of active substance (Table 1), the effectiveness of DE in the practical trials was worse, although we could not run an appropriate statistical analysis. We decided to use whole houses as replicates rather than house segments. Therefore, data are not conclusive for houses treated with Silicosec and Ewazid. An alternative would have been to combine product groups (natural vs. synthetic). But even then, only two houses would have been available as repeats for DE treatment, even though the development of the mite population proved to be similar. In order not to wrongly judge products here, we decided to be more reserved in our argumentation. The effectiveness could have been influenced due to the physical properties of the active substances, as the laboratory tests suggest. For a naturally derived product such as DE, efficacy might vary among production batches. Other relevant parameters such as concentration, particle size and adhesion were not collected in the context of this study.

Nevertheless, when Fossil Shield Instant White is used in the house, the mite population was kept very low over a period of 31 weeks and no colonies appeared, only individual mites. With the correct post-treatment of individual mite colonies, directly when they occur, it should be possible to get *D. gallinae* in the henhouse under control, at least for a longer period without the use of chemicals. In practice, it can be concluded that under the aspects of animal welfare, the use of DE requires earlier post-treatment compared to treatment with synthetic SiO_2_ products like Fossil Shield Instant White, to keep the mite population low.

The use of smaller amounts of material results in comparatively lower dust exposure and is therefore also safer for the professional user. This is becoming more important in the context of approval in the EU. For example, although the active ingredient SiO_2_ (DE, synonym Kieselguhr) is approved under the EU Biocide Directive, in November 2016 (Assessment Report 2016), it is stated that “the exposure of professionals to silicon dioxide DE during the large scale dusting in houses leads to an unacceptable risk”. This residual risk is due, among other things, to the fact that natural DE, unlike synthetic products, contains a smaller proportion of crystalline SiO_2_. The Assessment Report (2016) puts this at up to 0.1%. The reduction of dust exposure for the user by a considerably lower application rate is therefore of clear advantage.

It should also be noted that the effectiveness of the silica materials used is reduced at high humidity levels, such as those found in the chicken houses. This can be attributed to two parameters: (1) the water loss of the mites after damage to the epicuticle is reduced, and (2) the partly hydrophobic agents saturate with water and can no longer absorb lipids (Mewis and Ulrichs 2001). At least saturation is not a major problem with synthetic agents, as they are hydrophobic rather than hydrophilic and thus maintain their long-term efficacy. Therefore, companies selling natural DE-based products often blend in hydrophobic synthetic particles to ensure a longer-lasting effect.

Development of more comprehensive IPM regimens for *D. gallinae* should be facilitated by ongoing developments in monitoring, modeling of populations and management options with new combined materials to minimize the risk of resistance. Methods for monitoring mites in the occupied chicken house, the right application technology and formulations optimized for spot treatment are especially important criteria for future research. In practice, it has often proved to be advantageous to treat the surfaces in the cleaned houses with liquid formulations first. Spot treatments can then be carried out with powdery substances, applied electrostatically, in the occupied houses later to maintain low mite populations.
